# The Effect of Four Anaesthetic Protocols for Maintenance of Anaesthesia on Trans-Diaphragmatic Pressure in Dogs

**DOI:** 10.1371/journal.pone.0075341

**Published:** 2013-10-04

**Authors:** Kiriaki Pavlidou, Ioannis Savvas, Yves P. S. Moens, Dimitrios Vasilakos, Dimitrios Raptopoulos

**Affiliations:** 1 Anaesthesiology and Intensive Care Unit, Companion Animal Clinic, Faculty of Veterinary Medicine, Aristotle University of Thessaloniki, Thessaloniki, Greece; 2 Anaesthesiology and Perioperative Intensive Care, Veterinary University, Vienna, Austria; 3 Anaesthesiology and ICU Department, AHEPA University Hospital, Thessaloniki, Greece; The University of Manchester, United Kingdom

## Abstract

The diaphragm is the main inspiratory muscle and the main indicator of diaphragmatic contractility is the trans-diaphragmatic pressure (P_di_). The aim of this clinical study was to determine the effect of four different anaesthetic protocols on P_di_ in anaesthetized healthy dogs. Eighty client-owned dogs were recruited in this clinical study. All the animals received dexmedetomidine and morphine as premedication and propofol for induction. Anaesthesia was maintained with one of four protocols: isoflurane (I), isoflurane with CRI of propofol (IP), isoflurane with CRI of fentanyl (IF), and isoflurane with CRI of ketamine (IK). When the surgical plane of anaesthesia was achieved, two balloon catheters were inserted, one into the stomach and one into the mid-third of the oesophagus for P_di_ measurement. P_di_ value was the highest in groups I (14.9±4.7 mmHg) and IK (15.2±3.5 mmHg) and the lowest in groups IP (12.2±3.2 mmHg) and IF (12.0±5.9 mmHg). There was a statistically significant difference (p = 0.029) between groups IK and IF. PE’CO_2_ was statistically significantly higher (p<0.0005) in group IF (7.7±0.8 kPa) than in group IK (6.5±0.7 kPa). Isoflurane alone or isoflurane with ketamine for the maintenance of anaesthesia seem to better preserve the respiratory function and the diaphragmatic contractility than isoflurane with either propofol or fentanyl in dogs. Therefore, the use of isoflurane or isoflurane with ketamine may be of benefit when animals with respiratory problems have to be anaesthetized.

## Introduction

It is well known that general anaesthesia depresses respiratory function. Anaesthetic drugs interfere centrally with the regulation of ventilation and ultimately with the function of the respiratory muscles [1], [2]. The diaphragm is the main inspiratory muscle and contributes to the 2/3 of the actual tidal volume [3], [4]. The effects of many inhalation (enflurane, halothane, isoflurane, sevoflurane) and injectable (propofol, thiopentone) anaesthetics on the respiratory function and more specifically on diaphragmatic contractility have already been studied in animals and humans [5], [6], [7], [8], whereas few data exist on the influence of opioids and methylxanthines [9], [10]. In all these studies, the diaphragmatic contractility was evaluated experimentally by measuring the trans-diaphragmatic pressure (P_di_) after electrical stimulation of the phrenic nerves.

Volatile anaesthetic agents have varying effects on diaphragmatic activity. While enflurane depresses diaphragmatic function in a dose-dependent manner, halothane and isoflurane show little effect on it [7], [8], [11], [12] and sevoflurane has an intermediate effect [13], [14]. It has also been shown that halothane depresses P_di_ and the electrical activity of the diaphragm in spontaneously breathing dogs [5].

A clinical study in humans has shown that propofol may cause depression in the contractile properties of a fatigued diaphragm [15], but no clinical studies in animals exist on the effect of propofol on diaphragmatic contractility.

P_di_ measurement, during a maximum inspiratory effort, is considered to be a very good indicator of diaphragmatic contractility in humans [16], [17] and helps assessing patients with respiratory muscle weakness [18], [19]. P_di_ is defined as the difference between the intra-abdominal (P_abd_) and the intra-pleural (P_pl_) pressure [20], [21], [22]. However, the measurement of P_abd_ and P_pl_ is not straightforward under clinical conditions and alternative less invasive techniques have been considered, substituted the measurement of P_abd_ and P_pl_ by the measurement of the pressure in the stomach (P_gast_) and in the oesophagus (P_oes_), respectively [23]. This approach has been adapted for use in dogs allowing for the monitoring of P_di_ and hence of diaphragmatic contractility during anaesthesia in a clinical setting [24].

The aim of this clinical study was to investigate the effect of four different anaesthetic protocols for maintenance of anaesthesia on P_di_ in healthy canine patients. Our hypothesis was that the different anaesthetic protocols might have different effects on diaphragmatic contractility.

## Materials and Methods

### Ethics Statement

For this prospective, randomized, non-blinded clinical study, approval from the Ethics Committee of the Faculty of Veterinary Medicine, Aristotle University of Thessaloniki, Greece was obtained (Νr. 451/27-1-2009). All the dog owners were informed in detail about the study protocol and a signed written consent was taken.

### Animal Population

The study population was animals admitted to Companion Animal Clinic of the Faculty Veterinary Medicine of Thessaloniki for elective surgery (soft tissue or orthopedic). Exclusion criteria were status ASA III or higher, presence or history of any respiratory disease, abdominal and/or thoracic surgery, and the necessity to apply mechanical ventilation during surgery.

All animals underwent only non-abdominal/non-thoracic surgical procedures to avoid any effects of the atmospheric pressure in the open cavities and of the surgical manipulations on the P_oes_ and P_gast_. Only animals in lateral recumbency were included, in an attempt to minimize the effect of the body position on the P_oes_ and P_gast_. It has been found that diaphragmatic contractility is less affected by positioning dogs in lateral recumbency [25]. Obese animals were excluded from the study as it has been shown that obesity may decrease diaphragmatic contractility in humans [26], [27], [28]. It has been shown that in very small or large breed dogs, the introduction and proper placement of the balloon catheter into the stomach is difficult and the P_gast_ measurement inaccurate [24]. Therefore, medium size dogs were used in this study.

Eighty client-owned dogs (52 males, 28 females) were enrolled in the study. They were 1–10 (2.7±2.1) years (mean±standard deviation) old, medium sized, non-obese, and weighing 5–30 kg (17.0±8.3).

The animals were hospitalized in the Clinic for at least one day before the surgery. Pre-anaesthetic evaluation included physical examination, complete blood count, serum biochemistry (albumin, urea, creatinine, alkaline phosphatase/ALP, alanine aminotransferase/ALT, potassium, glucose) and thoracic/abdominal radiographs.

### Anaesthetic Protocol

All animals were fasted for eight hours, while they had free access to water for up to two hours before the premedication. On the day of surgery, the animals were premedicated with dexmedetomidine (Dexdomitor, Pfizer Greece) at 175 µg m^−2^, and morphine (Morphine sulfate, Famar SA Greece) at 0.1 mg kg^−1^, intramuscularly, and placed in a quiet room. Twenty minutes later, the cephalic vein was catheterized and the administration of Lactated Ringer’s solution (LR’s, Vioser Greece) at 10 ml kg^−1^ h^−1^ intravenously commenced. At this time, carprofen (Rimadyl, Pfizer Greece) was also administered at 4 mg kg^−1^ intravenously. Anaesthesia was induced with propofol (Propofol MCT/LCT, Fresenius, Fresenius Kabi Greece) intravenously to effect. An initial dose of 1–2 mg kg^−1^ was given, followed, if needed, by incremental doses of 0.5–1 mg kg^−1^ until endotracheal intubation could easily be performed. Anaesthesia was maintained with one of the four protocols described below. All animals were breathing spontaneously. Fresh gas (100% oxygen) flow was delivered at 1.5 L min^−1^ through a circle rebreathing system. Throughout the whole procedure, heart rate (HR), respiratory rate (RR), non-invasive mean arterial blood pressure (MAP) (with the cuff placed around the forelimb), end-tidal carbon dioxide partial pressure (PE’CO_2_) and end-tidal isoflurane fraction (FE’iso) were constantly monitored (Datex-Ohmeda S/5, GE Healthcare, Helsinki, Finland) and recorded every 5 minutes. In case of a PE’CO_2_ higher than 8.5 kPa, artificial ventilation was applied and the measurement of P_di_ was cancelled. During surgery, all the animals were placed in lateral recumbency, either left or right.

### Experimental Groups

The animals were randomly allocated into four groups according to computer generated random numbers, which differed only in the protocol for the maintenance of anaesthesia. Four different maintenance protocols were used: isoflurane (Isoflurane, Merial Italy) (n = 20) (group I), isoflurane with CRI of propofol at 0.1 mg kg^−1^ min^−1^ (n = 20) (group IP) [29], [30], [31], isoflurane with CRI of fentanyl (Fentanyl, Janssen-Cilag Greece) at 0.2 µg kg^−1^ min^−1^ (n = 20) (group IF) [32], [33], and isoflurane with CRI of ketamine (Imalgene 1000, Merial Italy) at 0.2 mg kg^−1^ min^−1^ (n = 20) (group IK) [30]. In group IK, midazolam (Dormicum, Roche Greece) at 0.3 mg kg^−1^ was also administered intravenously, just after the end of the ketamine CRI and, irrespective of the duration of the surgery, after the end of P_di_ measurements. Furthermore, if any clinically significant change in the haemodynamic and respiratory parameters (more than 10% deviation from the initial measurements) was observed intra-operatively in any animal of the groups, FE’iso would be increased by increasing the dial setting of the vaporiser. Rescue analgesia was deemed necessary when the increase (up to 2.5%) in FE’iso was inadequate and fentanyl would be given IV, especially in the animals of groups I, IP and IK. These animals would have been excluded from the study.

### Trans-diaphragmatic Pressure Measurement

P_di_ was measured under the same anaesthetic level in all animals. Clinical signs along with electronic monitoring readings were used in order to determine the anaesthetic depth. Such signs were lack of reflexes, presence of adequate muscle relaxation, and a lack of physiological response to surgical stimulation characterized by less than 10% change in HR, RR and MAP during surgical stimulation.

Two 90 cm long oesophageal balloon catheters (Esophageal Balloon Catheter Set, CooperSurgical Company, Trumbull, USA) with guide wires were used for the P_di_ measurement. The balloon was localized close to the distal end of the catheter and it had a capacity of 3 ml of air.

When a surgical plane of anaesthesia was achieved, the two balloon catheters were introduced orally. The size of the balloon catheters was the same in all animals. However, as the body size of the animals was not the same, it proved helpful to mark the catheter in advance until the point that the balloon should be inserted into the stomach or in the mid-third of the oesophagus. It has been found that the place of the lower oesophageal sphincter can be determined after subtracting 5 cm from the pre-measured distance between the external length from lower jaw incisor tooth to the anterior border of the head of the 10th rib through the ankle of the mandible [34].

Using the above anatomical landmarks, the balloon of the first catheter was introduced into the stomach for the measurement of P_gast_ and the distal end of the second catheter was positioned in the mid-third of the oesophagus for the measurement of P_oes_. The correct positioning of the two catheters was confirmed by the observation of the respective pressure tracings on the computer screen: a positive deflection (increase in pressure) during each inspiration was an indication of the correct intra-gastric position of the balloon of the catheter, whereas a negative deflection (decrease in pressure) at inspiration confirmed oesophageal placement of the balloon of the second catheter. The catheters were secured in place by fixing them to the endotracheal tube. Following the removal of the guide wires, they were connected to the pressure transducers and the balloons were inflated with 0.5–1 ml of air.

The proximal end of the catheters was connected to a pressure transducer. The analog signal was digitized in a dedicated device and the data were saved to a computer. Custom made software allowed display of the pressure profile on the computer screen (Pressure Monitoring system Buzzer-II, Michael Roehrich, Austria). The pressure transducers were zeroed to the atmospheric pressure during assembly of the device prior to each measurement.

All pressure measurements were sampled with a rate of 10 Hz. For the measurement of P_di_, it was necessary to obtain the maximum deflections of P_gast_ and P_oes_ during a respiratory cycle. To achieve this, the endotracheal tube was disconnected from the anaesthetic circuit and tightly closed with a thumb at the end of expiration, so that the animal was forced to breath against a completely closed airway (modified Mueller’s manoeuvre) [24]. This manoeuvre was applied for three consecutive respiratory cycles, every five minutes for the whole duration of the measurements.

P_gast_ and P_oes_ were continuously monitored for 90 minutes. Thus, one set (of three measurements each) of both gastric and oesophageal pressure values were obtained, every five minutes for 90 minutes, irrespective of the duration of the surgical procedure, and P_di_ values were calculated in total at 19 time points in each animal.

### Data Processing and Statistical Analysis

**Figure 1:**
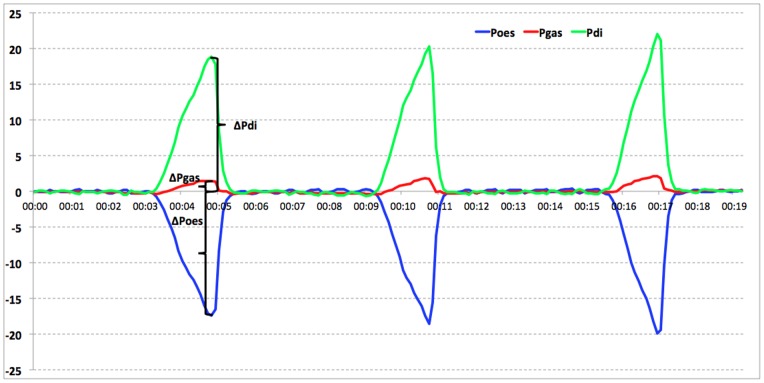
Three consecutive respiratory cycles after the application of the modified Mueller’s manoeuver. During inspiration the intra-oesophageal pressure becomes negative (ΔP_oes_) and the intra-gastric pressure positive (ΔP_gas_). Their difference is the P_di_ (P_di_ = ΔP_gas_-ΔP_oes_). The three P_di_ values in each time point were averaged to one value.

All the data were saved in a spreadsheet and analysed with a signal analysis software (Qtiplot, MicroCal, Northampton, Massachusetts, USA). From the data for each pressure (gastric or oesophageal), a positive curve for gastric pressure and a negative curve for oesophageal pressure with three peaks and three valleys, respectively, were drawn. The baseline of gastric and oesophageal curves was zeroed. In every set of measurements, the P_di_ value was calculated (difference between P_gast_ and P_oes_). Then, the three highest values (peaks) from the P_di_ graph of each 3-breaths cycle were averaged and one single value of P_di_ was obtained for every time point (Figure 1). These averaged values of P_di_ in each time point (19 values for the total time of 90 minutes) were used to calculate the area under the curve (AUC) of P_di_, in each animal, using the trapezoid method [35]. The AUCs of the other measured variables (HR, RR, MAP, PE’CO_2_, FE’iso) were also calculated. The AUCs were then standardised by the duration of measurements (in minutes). The Shapiro-Wilk test of normality was used to evaluate the normality of data. All the measurements were analysed with a General Linear Model with one between-subject factor with four levels and one within-subject factor (time). Then ANOVA with least significant difference (LSD) correction was used to reveal any differences among the groups. p<0.05 was considered to be statistically significant.

## Results

The four groups were homogenous regarding the age (p = 0.793) and the body weight of the animals (p = 0.442), and the total administered dose of propofol for induction (p = 0.129). The mean±standard deviation of the age was 2.5±1.9 in group I, 2.8±2.2 in group IP, 2.6±2.6 in group IF and 2.7±1.9 in group IK, of the weight 17.2±9.8 in group I, 18.4±7.8 in group IP, 16.8±8.1 in group IF and 15.5±7.6 in group IK and of the administered dose of propofol 2.8±2.3 in group I, 2.3±0.9 in group IP, 2.7±2.0 in group IF and 2.3±0.7 in group IK. Descriptive statistics of all the haemodynamic and respiratory parameters and statistically significant differences among the four groups are presented in Table 1.

**Table 1:** Mean±standard deviation of the haemodynamic and respiratory parameters in the four groups.

variable	Group I (n = 20)	Group IP (n = 20)	Group IF (n = 20)	Group IK (n = 20)
HR (min^−1^)	99.8±17.2^IF,IK^	90.9±13.8	82.1±12.0^I^	87.6±15.4^I^
MAP (mmHg)	75.7±11.6^IP,IK^	87.0±11.9^I^	72.6±13.2^IP,IK^	84.7±15.3^I,IF^
RR (min^−1^)	9.3±3.3	8.4±3.3^IF^	11.3±4.3^IP^	9.2±3.3
PE’CO_2_ (kPa)	6.7±0.8^IP,IF^	7.5±1.0^I,IK^	7.7±0.8^I,IK^	6.5±0.7^IP,IF^
FE’iso (%)	1.83±0.49^IP,IF, IK^	1.19±0.28^I,IK^	1.41±0.38^I^	1.52±0.22^I,IP^

* Superscripts indicate groups that differ statistically significantly from column group.

HR: heart rate, MAP: mean arterial pressure, RR: respiratory rate, PE’CO_2_: end-tidal carbon dioxide partial pressure, FE’iso: end-tidal isoflurane fraction.

On the basis of the fact that no clinically significant change in the haemodynamic and respiratory parameters (more than 10% deviation from the baseline) occurred intra-operatively, the level of analgesia in the four groups seemed to be sufficient and no rescue analgesia was deemed to be necessary on any case.

### Trans-diaphragmatic Pressure (P_di_)

**Figure 2:**
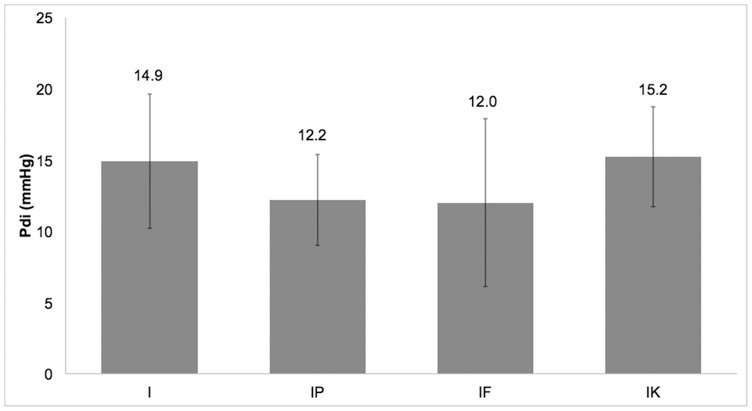
Mean±standard deviation of trans-diaphragmatic pressure (P_di_) in the four groups (I, IP, IF, IK).

The highest mean value of P_di_, 15.2±3.5 mmHg, was observed in group IK (Figure 2). Groups I, IF, and IK differed significantly (p = 0.044 - I compared with IF, p = 0.029 - IF compared with IK). Furthermore, there was a statistically significant difference between groups IP and IK (p = 0.038) (Table 2). No interaction between treatment groups and time was detected (p = 0.511).

**Table 2:** Mean±standard deviation of the trans-diaphragmatic pressure (P_di_) in the four groups.

variable	Group I (n = 20)	Group IP (n = 20)	Group IF (n = 20)	Group IK (n = 20)
P_di_ (mmHg)	14.9±4.7^IF^	12.2±3.2^IK^	12.0±5.9^I,IK^	15.2±3.5^IP,IF^

* Superscripts indicate groups that differ statistically significantly from column group.

P_di_: trans-diaphragmatic pressure.

### Mean Arterial Pressure (MAP)

MAP was significantly higher in group IK than in group IF (p = 0.006). Moreover, there was a statistically significantly higher MAP in group IP than in group I (p = 0.011), and a statistically significantly lower MAP in group I than in group IK (p = 0.043) (Table 1).

### End-tidal Carbon Dioxide Partial Pressure (PE’CO_2_)

**Figure 3:**
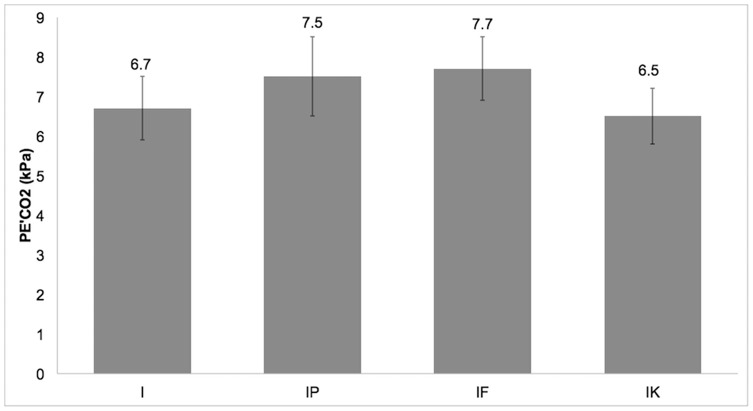
Mean±standard deviation of end-tidal carbon dioxide partial pressure (PE’CO_2_) in the four groups (I, IP, IF, IK).

PE’CO_2_ was higher in groups IP and IF than in groups I and IK (Figure 3). PE’CO_2_ differed significantly between groups I and IP (p = 0.004), I and IF (p = 0.001), IP and IK (p<0.0005), and IF and IK (p<0.0005) (Table 1).

### End-tidal Isoflurane Fraction (FE’iso)

FE’iso was lower in group IP than in groups I, IF and IK. FE’iso differed statistically significantly between groups I-IP (p<0.0005), I-IF (p = 0.001), I-IK (p = 0.010) and IP-IK (p = 0.007) (Table 1).

## Discussion

The present clinical study was designed to investigate the effect of four anaesthetic protocols for maintenance of anaesthesia on diaphragmatic contractility in dogs. The evaluation of diaphragmatic contractility was based on P_di_ measurement with balloon catheters. To the best of the authors’ knowledge, this is the first presentation of P_di_ measurements in a non-fatigued diaphragm in dogs during anaesthesia. P_di_ values ranged from 12.0±5.9 mmHg to 15.2±3.5 mmHg in dogs under the anaesthetic protocols used. Another finding of this clinical study was that P_di_ was higher in groups I and IK than in groups IP and IF.

The present study was not fully blinded. The anaesthetist was aware of the chosen protocol in order to recognize and treat the side effects of the drugs and to maintain a similar depth of anaesthesia in all animals. This person might have been able to see the different pressure tracings but was unaware of the numerical values. Moreover, the investigator who was monitoring the waveforms of the diaphragmatic pressure was not aware of the anaesthetic protocol that was used.

Diaphragmatic contractility and evaluation of P_di_ with balloon catheters is a subject, which has been investigated in fatigued, intact diaphragm after electrical stimulation of the phrenic nerves in human patients [16], [21], [36]. It is reported that the P_di_ value during a normal sniff is more than 58.84 mmHg in men and more than 51.49 mmHg in women [37]. In veterinary medicine, the effect of different drugs on diaphragmatic contractility has also been studied by measuring P_di_ in a fatigued diaphragm [7], [8], [11], [13], [14].

The modified Mueller’s manoeuvre was applied for three consecutive respiratory cycles, so that three values could be obtained every five minutes and a more accurate mean of the three pressures be calculated. In human medicine, Mueller’s manoeuvre is the most commonly used occlusion test. During the application of this technique, the maximum increase in P_gast,_ and the maximum decrease in P_oes_ are observed, and the maximum deflection of P_di_ can be calculated in inspiration. In this clinical study, it was necessary to modify the Mueller’s manoeuvre with the close of the endotracheal tube with the thumb, as it has been applied only to awake humans.

However, the application of this occlusion test may affect respiratory function, as post-obstructive pulmonary edema (POPE) has been developed under these circumstances in humans. Prolonged inspiration against a fixed obstruction, which resulted in pulmonary edema, was firstly noted in experimental dog models exposed to resistive load in 1927 [38]. The application of the Mueller’s manoeuvre can also cause POPE. Although POPE can be developed in a few minutes in patients under anaesthesia, there is no reference to the development of POPE in dogs under anaesthesia in clinical setting. Additionally, POPE has not been reported in any clinical study in awake patients for the P_di_ measurement with the application of the Mueller’s manoeuvre [20], [21], [22], [23]. Clinical signs of POPE are tachypnoea, tachycardia and rhonchi, and chest radiographs may confirm pulmonary edema [39]. It may be concluded that the possibility of an animal to develop POPE is very low. Interestingly, clinical signs of POPE were not observed in any animal for up to 24 hours post-anaesthesia in the present study.

The premedication was the same in all groups so that the same pre-anaesthetic level of sedation would be achieved. The choice of dexmedetomidine and morphine as premedicants was based on their sedative-analgesic effects. Morphine does not seem to affect the contractility of a fatigued diaphragm [10], [40], [41], while there are no published data for the effect of α_2_-agonists on diaphragmatic contractility. Carprofen was also used for additional analgesic effects. It has been shown to provide adequate analgesia in orthopaedic procedures in dogs, especially when it is given prior to surgery [42], [43]. However, its effect on diaphragmatic contractility is not known.

There is little information about the influence of propofol on diaphragmatic contractility in the intact diaphragm. Propofol affects P_di_ in a dose dependent manner in humans. In particular, the smaller the dose of propofol is the less effect on diaphragmatic contractility can be observed [15]. In the present study, propofol seemed to produce profound depression of the diaphragmatic contractility.

Fentanyl, as an opioid, causes a direct effect on brain-stem respiratory centers, leading to a dose-dependent depression of the respiratory system. This effect is added to the respiratory effect of isoflurane and may explain why the values of PE’CO_2_ were higher in group IF than in the other groups [44] (Table 1, Figure 3). In the present study, the lowest P_di_ value was observed in group IF. The administration of opioids (morphine, fentanyl) can increase the abdominal muscle activity and, thus, P_abd_. In particular, it was found that the administration of fentanyl in patients during laparoscopy increased the contraction of the diaphragm during inspiration and as a result P_abd_ and P_gast_ were increased [45]. However, it has been found that the administration of fentanyl at 1 µg kg^−1^ during capnoperitoneum in spontaneously breathing dogs did not increase P_abd_ [46].

Ketamine appears to have less profound effects on the respiratory muscles than other anaesthetics agents, as it seems to preserve muscle tone [47], [48]. In *in vivo* studies there are few data about the influence of ketamine alone or in combination with isoflurane on diaphragmatic contractility [12], [49]. The effect of ketamine, thiopental and propofol on muscle contractions in rat diaphragm *in vitro* has been investigated and it has been found that ketamine could preserve the muscle tone [50].

Another factor that may affect diaphragmatic contractility is diaphragmatic blood flow. Mean arterial blood pressure is an index of peripheral blood flow and, therefore, may be used as an indicator of diaphragmatic blood flow. Diaphragmatic blood flow decreases when the MAP decreases below a critical pressure of 60 mmHg in dogs [51], [52]. As MAP was significantly higher in group IK than in groups I and IF in the present study, it could be argued that the preservation of blood flow to the diaphragm may have contributed to the higher P_di_ values observed in group IK. However, MAP was lower in group IK than in group IP but without statistically significant difference.

Notably, in the present study the CRI of the drugs was maintained in a fixed infusion rate, and any necessary change in the depth of anaesthesia was achieved by adjusting the FE’iso. Therefore, the FE’iso was not kept stable in the four protocols during the measurements. It was anticipated that on basis of the weak effect of isoflurane on diaphragmatic contractility [11], [12], small changes in FE’iso would have little effect on P_di_, compared with the effect of the different infused drugs. And, as it has already been mentioned, in no case the administration of rescue analgesia was deemed to be necessary.

A limitation of the study is that plasma levels of the injectable drugs used for the maintenance of anaesthesia were not measured. It is likely that the plasma levels increased gradually during the measurement, especially in the beginning of the CRI, according to the specific pharmacokinetic properties of each drug. This may have influenced P_di_ over time. However, clinical experience suggests that drug effects appear rapidly after commencement of CRI, even without preceding bolus loading. Moreover, it seems that in this study the drug effects were not influenced by time; the only effect was the drugs used and this effect can be seen from the beginning of the CRI, i.e. about 5 minutes after the start of the infusion. Additionally, the haemodynamic and respiratory parameters did not change significantly over the measurement period, suggesting a minimal effect of time on the measured variables.

## Conclusion

Diaphragmatic contractility and hence respiratory function might be better preserved during the maintenance of anaesthesia with isoflurane alone or in combination with ketamine infusion compared to the combination of isoflurane with propofol or fentanyl on the basis of P_di_ results. This might be of benefit when animals with respiratory problems have to be anaesthetized. However, further studies are needed in order to investigate the effect of various drugs used for premedication, and induction or maintenance of anaesthesia on trans-diaphragmatic pressure and diaphragmatic contractility in animals in different physical conditions.
